# The Impact of Body Resistance Training Exercise on Biomedical Profile at High Altitude: A Randomized Controlled Trial

**DOI:** 10.1155/2021/6684167

**Published:** 2021-06-01

**Authors:** Gaffar Sarwar Zaman, Mohammed Abohashrh, Irshad Ahmad, Ayed A. Dera, Mastour S. Alshahrani, Irfan Ahmad, Mohammad Mahtab Alam, Syed Esam Mahmood, Nasrin Mansuri, Safia Irfan, Forhad Akhtar Zaman

**Affiliations:** ^1^Department of Clinical Laboratory Sciences, College of Applied Medical Sciences, King Khalid University, Abha Zip code: 62521, Saudi Arabia; ^2^Department of Basic Medical Sciences, College of Applied Medical Sciences, King Khalid University, Abha Zip Code: 62521, Saudi Arabia; ^3^Department of Medical Rehabilitation Sciences, College of Applied Medical Sciences, King Khalid University, Abha Zip Code: 62521, Saudi Arabia; ^4^Department of Family and Community Medicine, College of Medicine, King Khalid University, Abha Zip Code: 61421, Saudi Arabia; ^5^Department of Physiology, College of Medicine, King Khalid University, Abha Zip Code: 62521, Saudi Arabia; ^6^Department of Community and Family Medicine, All India Institute of Medical Sciences (AIIMS), Pin: 781032, Guwahati, India

## Abstract

**Background:**

Obesity causes different diseases, eventually. In our study, the results of resistance exercises were examined on selected biochemical markers in Abha City, Saudi Arabia, which is at the height of 2,270 meters above sea level.

**Methods:**

A randomized controlled research was conducted with 60 participants equally divided into three groups, 20 subjects in each group: group 1 was composed of obese people who received resistance training exercise, group 2 was composed of the obese control group who did not receive resistance training exercise, and group 3 was composed of normal individuals who received resistance exercise training. The resistance exercises were done in the 6th and 12th weeks. Biochemical blood tests were done.

**Results:**

Comparing to the control group, glucose decreased very little with insulin also showing little difference. It has been seen that TC, TG, and LDL reduced to a reasonable extent after resistance exercise, while HDL was increased (*p* ≤ 0.01). Plasma urea and creatinine showed no differences. Interleukin-6 and leptin decreased significantly (*p* ≤ 0.01), while there was a significant elevation in adiponectin and testosterone (*p* ≤ 0.01) once comparing group 1 with group 2 and group 3.

**Conclusion:**

We have seen that resistance exercise helps in reducing lipid profile which will result in a decrease of the cardiac and related risk factors when conducted in obese patients in high-altitude regions. Also, alterations of the levels of interleukin-6, leptin, adiponectin, and testosterone showed that resistance exercise is of benefit and favourable in obese persons in high-altitude regions, which can also pave the way for added development of drugs related to the above parameters.

## 1. Introduction

Obesity has long been a subject of research for the various diseases it causes in the long run. It was noticed by Al-Nozha et al. in 2005 that obesity and overweight are escalated in Saudi Arabia (KSA) with a countrywide prevalence of obesity of 35.5% [1]. Over the past few years, KSA has become the country with one of the major prevalence rates in terms of overweight and obesity [2]. Previous studies concerning prevalence of obesity in the KSA specify an escalating trend in obesity and overweight, which are the main sources of a number of other diseases, including diabetes, hypertension, obstructive sleep apnea, hyperlipidemia, and osteoarthritis [3].

It has been evidenced that aerobic exercise attribute elevates the glucose intake due to the augmented insulin sensitivity in the receptor of muscle tissue. These advantageous differences were noticed both in elevated protein expression of a receptor for insulin and its parts, Insulin receptor substrate 1 and Insulin receptor substrate 2 (IRS-1 and IRS-2) [4]. However, few researches acquainted us with details and particulars on the consequences of resistance exercise (RE) on the amount of glucose in the blood and on the enhancement of skeletal muscle insulin sensitivity [5]. A short while back, high-intensity intermittent exercise has been accentuated for the intention of weight depletion and downgrading the atherogenic index [6, 7]. Contradictory outcomes have been reported that advocate these for gaining benefit in the lipoprotein levels, lipid, and hypertension. These types of exercise have shown a remarkable upgradation in the cardiometabolic profile [8]. Various epidemiological researches have depicted that physically active individuals have less incidence of coronary heart disease juxtaposed to sedentary people. Exercise instigates acute increment in post heparin lipoprotein lipase, which, in turn, accompanies amplifying the clearance of triglycerides, and declines the clearance of high-density lipoprotein (HDL) constituents [9]. It has been seen that well-organized physical activity has a number of positive benefits on blood lipid profile. Cross-sectional studies have clearly depicted that well-trained endurance athletes have low levels of triglycerides (TG) and low-density lipoprotein (LDL) amounts and increased HDL levels when compared to untrained individuals [10]. Exercise escalates energy disbursement, the consequence of which is enhancing the breakdown of amino acid, particularly the oxidation of certain amino acids [11]. Moderate exercise encourages an elevation in protein breakdown and urea and creatinine metabolic waste elimination. Researchers have shown that short consequences of activities lead to a decrease in cholesterol (TC), LDL, TG, interleukin-6 (IL-6), and leptin and an increase in testosterone, adiponectin, and HDL level [12].

Researches have shown many positive correlations between body weight and resistance to insulin; moreover, the probability of developing all the metabolic idiosyncrasy is strongly interconnected with resistance to insulin [13]. Also, the lack of data from many parts of KSA, especially from the Aseer region, which is located at a high altitude, regarding the attributes of various types of exercise in the action on various biochemical parameters makes it necessary for such an investigation. Since very few researches have been conducted in high-altitude regions regarding glucose profile, kidney profiles, lipid profile, adipokines, testosterone, etc., this extensive study will help in concluding these parameters and will help future physicians to play their role more efficiently and adequately. Hence, our main aim was to have a detailed examination on the relationships between obesity and exercise-related physical activity in adults, with exceptional emphasis on the interaction between obesity and factors relating to diabetes, kidney profile, lipid profile, interleukin-6, leptin, adiponectin, and testosterone, in high-altitude regions.

## 2. Methods

### 2.1. Study Design

This is randomized controlled research conducted in Abha, Aseer state in the Kingdom of Saudi Arabia, involving male gender within the age bracket of 35–60 years.

### 2.2. Recruitment of Patients

The participants were enlisted through individualized correspondence. The participants were separated into three groups encompassing 20 in each group: group 1 was composed of obese people who received resistance training exercise, group 2 was composed of the obese control group who did not receive resistance training exercise, and group 3 was composed of normal individuals who received resistance exercise training.

### 2.3. Dropouts

Initially, 25 participants in each group were chosen for the study. But due to noncommitments, noncompliance with exercise protocol, or other issues, 5 subjects were considered as dropouts, and only 20 in each group were retained till the end of the study.

### 2.4. Exercise Procedure

Subjects in group 1 and group 3 were given resistance exercise training of 50–70% of 1RM (Maximum Repetition) 3 days per week for a period of 12 weeks. Resistance exercises in each session were ten repetitions with three sets for the first six weeks; one repetition was added in every week for the next six last weeks after each set 30-second rest was given. Ten-minute warm-up, 5-minute self-stretching, and 30-minute work-out followed by a 5-minute cool-down session were performed. Exercise sessions were done indoor in the physical therapy exercise gym at noontime 1 hour before meal under specialist supervision. Vital sign parameters were examined before and after the session. The main work-out session was focused on large muscles of the upper and lower extremities, spinal extensors, and abdominal muscles: exercises for the upper limb (shoulder push, push-ups, and side pull-ups and down), lower limb (squats, leg press, and leg curl), spinal extensors (upper and lower spinal back rising against gravity), and abdominals (sit-ups for rectus and oblique abdominals). Group 2 did not receive any resistance exercises.

### 2.5. Inclusion and Exclusion Criteria

Inclusion of participants was based on age (35-60 years); participants who were willing to go through the explained exercises were duly selected. The yardstick for exclusion was entrenched as follows: well-established dyslipidemia and diagnosis interconnected with secondary dyslipidemia. Secondary sources of dyslipidemia encompassed infectious diseases, established family history of familial hypercholesterolemia, metabolic syndrome, and renal failure; subjects with poor or irregular compliance with the exercise protocol were excluded from the final result analysis. Also, dropouts due to various reasons were excluded from the study.

### 2.6. Sample Collection

After an overnight fast of about 10-12 hours, approximately 5 ml of whole blood was taken aseptically by venipuncture from all the subjects in two vials—a sodium fluoride vial and an ethylenediaminetetraacetic acid (EDTA) vial, between 7:30 and 9:30 am. The same was done 2-3 hrs postprandially. The samples were then centrifuged to collect the serum which was stored at -20°C. The categories of Body Mass Index (BMI) were defined in accordance with the World Health Organization criteria [14].

### 2.7. Biochemical Assays

TC was ascertained by the cholesterol oxidase phenol 4-aminoantipyrine peroxidase (CHOD-PAP) method, which is an enzymatic colorimetric test for cholesterol with a lipid clearing factor, utilizing the kit from Human company, Germany [15]. The enzymatic colorimetric test for the quantitative determination of TG in human plasma was done by the peroxidase-coupled method for the colorimetric determination of serum triglycerides as delineated by McGowan et al. [16]. The evaluation of HDL was accomplished utilizing the technique arrogated in Tietz et al. [17] while the technique of Assman et al. [18] was espoused in the verification of LDL (in which precipitation of LDL with polyvinyl sulphate is the main), and very-low-density lipoprotein (VLDL) was delineated utilizing the technique of Friedwald et al. [19]. Glucose estimation was done by the GOD-PAP enzymatic colorimetric method (without deproteinization); urea estimation was done by the modified Barthelot reaction method, and creatinine estimation was done by the Jaffe reaction, which is a photometric colorimetric test for kinetic measurements (without deproteinization). All of the above were estimated utilizing the Human kit company from Germany, using the spectrophotometer. Plasma levels of the hormones of curiosity were quantitatively measured by enzyme-linked immunosorbent assays (ELISA). Prepackaged ELISA kits were used to estimate insulin, leptin, adiponectin, interleukin-6, and testosterone. Simultaneous binding of human insulin by two monoclonal antibodies is the basis of the insulin ELISA test, one being immobilized on microwell plates and the other being conjugated with horseradish peroxidase (HRP). “Sandwich” ELISA is utilized in the immunoassay for leptin where the target protein (antigen) is bound in the format of a “sandwich” by the capture of antibodies coated to each well-bottom and the secondary detection antibodies added subsequently by the investigator. A specific antibody for human adiponectin is employed in the adiponectin assay. Pipetting of the standards and samples is done into the wells, and the existing adiponectin in the sample is attached to the wells by the immobilized antibody. After washing of the wells, biotinylated anti-human adiponectin antibody is added. After washing away unbound biotinylated antibody, HRP-conjugated streptavidin is pipetted to the wells. Washing of the wells is done, and a TMB (3,3′,5,5′-tetramethylbenzidine) substrate solution is added to develop color in proportion to the amount of adiponectin bound. Utilization of the double-sandwich ELISA technique is done for estimation of interleukin-6. Human IL-6 monoclonal antibody is precoated to the wells, and the detecting antibody is biotin labelled polyclonal antibody. The ELISA for testosterone was mainly done by utilizing the principle of competitive interaction of hormone-enzyme conjugate and testosterone for a limited number of immobilized monoclonal anti-testosterone antibodies. Thus, the amount of bound hormone-enzyme conjugate was inversely proportional to the concentration of testosterone in the specimen.

### 2.8. Ethical Approval

The ethical approval for this particular research was taken from the Research Ethics Committee of King Khalid University, KSA, vide reference number REC # 2018–05–10.

### 2.9. Data Analysis

All statistical data were scrutinized and interpreted utilizing the Statistical Package for the Social Sciences software (SPSS 23.0; Chicago, IL). The gathered data are delineated as the mean ± SD (standard deviation). Analysis of variance (ANOVA) with the Bonferroni test were also utilized to address the issue arising from multiple comparisons. A *p* ≤ 0.01 level of significance was considered.

## 3. Results

### 3.1. Effects of Resistance Exercises on Glucose and Insulin Level

**Figure 1:**
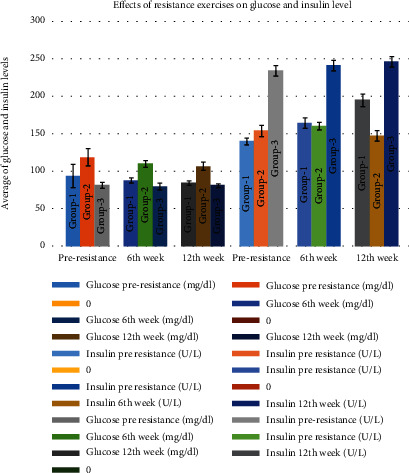
Effects of resistance exercises on glucose and insulin level.

Plasma glucose seemed to slightly decrease in the patients of group 1 from 93.87 ± 15.48 mg/dl (in the preresistance group) to 88.07 ± 3.5 mg/dl (in the 6th week) and to 84.53 ± 2.95 mg/dl (in the 12th week). There seemed to be little or no change in group 2 (119.07 ± 11.47 mg/dl in the preresistance group, 110.53 ± 4.56 mg/dl in the 6th week, and 106.87 ± 5.48 mg/dl in the 12th week). Also, there seemed to be no change in group 3 regarding glucose (81.73 ± 3.71 mg/dl in the preresistance group, 80.07 ± 4.75 mg/dl in the 6th week, and 81.33 ± 2.67 mg/dl in the 12th week) (Figure 1). Insulin also seems to be in the normal range, although it appears to be slightly lower in the 2nd group of the pre-, 6th week, and 12th week (154.25 ± 7.63 U/l, 160.45 ± 5.19 U/l, and 147.40 ± 6.83 U/l); in the 1st group, it seemed to increase from 140.18 ± 4.40 U/l in the preresistance group to 164.50 ± 7.12 U/l in the 6th week and then up to 195.10 ± 8.59 U/L in the 12th week. The 3rd group showed very minute changes (Figure 1).

There is no significant difference between glucose in the preresistance, 6th week, and 12th week between the three groups (Table 1). Also, there is no significant difference between the groups when compared in various weeks as per the Bonferroni test (Table 2). There is a considerable difference between insulin in the preresistance and insulin in the 12th week, as per the Bonferroni test (*p* ≤ 0.01) (Table 2).

### 3.2. Effects of Resistance Exercises on Lipid Profile

**Figure 2:**
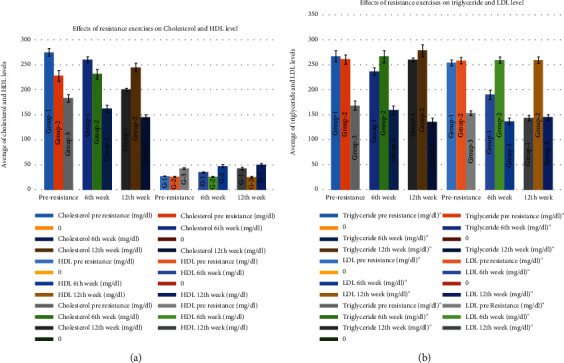
Effects of resistance exercises on lipid profile. (a) Effects of resistance exercises on cholesterol and HDL. (b) Effects of resistance exercises on triglyceride and LDL.

Triglyceride was higher in the pre- 1st group (267.50 ± 11.42 mg/dl) and 2nd group (260.92 ± 9.44 mg/dl), 6th week 1st group (237.10 ± 7.86 mg/dl) and 2nd group (266.97 ± 12.20 mg/dl), and 12th week 2nd group (278.68 ± 12.39 mg/dl). It was comparatively normal to borderline in the 3rd group in the pre-, 6th week, and 12th week (168.53 ± 9.54 mg/dl, 159.72 ± 8.81 mg/dl, and 135.43 ± 8.41 mg/dl, respectively) (Figure 2(a)). There seemed to be lowering of the LDL level from the pre- to the 12th week in the 1st group (254.27 ± 6.14 mg/dl to 190.40 ± 9.41 mg/dl in the 6th week and 143.67 ± 5.32 mg/dl in the 12th week), but no such lowering was seen in the 2nd group (258.83 ± 67.92 mg/dl, 259.42 ± 6.46 mg/dl, and 268.83 ± 7.86 mg/dl); the 3rd group showed very minimal reduction (153.67 ± 4.93 mg/dl in the pre-, 137.20 ± 6.71 mg/dl in the 6th week, and 126.72 ± 5.75 mg/dl in the 12th week) (Figure 2(a)). The mean ± SD for cholesterol in the pre- and 6th week for group 1 (274.9 ± 7.76 mg/dl and 260.5 ± 5.54 mg/dl) and all weeks of group 2 (228.20 ± 11.07 mg/dl, 231.50 ± 9.54 mg/dl, and 244.90 ± 8.76) was higher than normal, but for the 3rd group, almost all were in the normal range (183.32 ± 7.77 mg/dl, 162.45 ± 7.17 mg/dl, and 145.20 ± 5.09 mg/dl) and showed a decline as time passed (Figure 2(b)). The HDL was comparatively lower in the pre- 1st group (27.53 ± 0.5 mg/dl), pre- 2nd group (26.27 ± 0.9 mg/dl), 6th week 1st group (35.33 ± 1.17 mg/dl) and 2nd group (26.13 ± 0.95 mg/dl), and also in the 12th week 2nd group (25.80 ± 1.95 mg/dl). However, HDL was borderline in the pre- 3rd group (42.87 ± 2.09 mg/dl), 6th week 3rd group (47.80 ± 2.98 mg/dl), and 12th week 1st group (42.93 ± 2.4 mg/dl) and normal in the 12th week 3rd group (50.53 ± 2.7 mg/dl) (Figure 2(b)).

A significant difference is seen in cholesterol, HDL, LDL, and triglyceride, as can be seen from the above results, between the groups in the ANOVA tests, and also between time as can be seen from the Bonferroni test (*p* ≤ 0.01) (Tables 1 and 2).

### 3.3. Effects of Resistance Exercises on Kidney Profile

**Figure 3:**
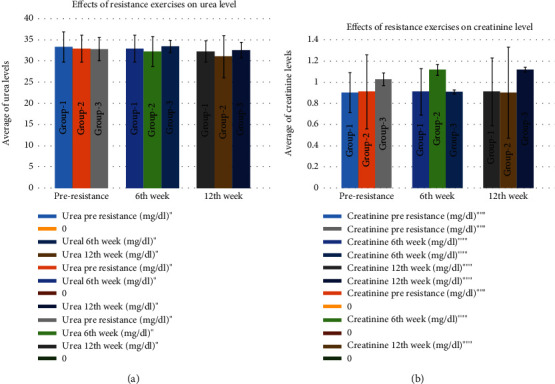
Effects of resistance exercises on kidney profile. (a) Effects of resistance exercises on blood urea. (b) Effects of resistance exercises on blood creatinine.

Urea remained in the normal range in all the participants. Urea in the 1st group was 33.33 ± 3.57 mg/dl in the pre-, 32.93 ± 3.17 mg/dl in the 6th week, and 32.27 ± 2.540 mg/dl in the 12th week. In the 2nd group, it was 32.93 ± 3.17 mg/dl in the pre-, 32.27 ± 3.54 mg/dl in the 6th week, and 31.07 ± 4.99 mg/dl in the 12th week (Figure 3(a)). The creatinine level, in the 1st group, was 9.034 ± 0.19 mg/dl in the pre-, 9.12 ± 0.22 mg/dl in the 6th week, and 9.12 ± 0.32 mg/dl in the 12th week. For the 2nd group, it was 9.12 ± 0.35 mg/dl in the pre-, 1.12 ± 0.052 mg/dl in the 6th week, and 9.03 ± 0.43 mg/dl in the 12th week. The 3rd group also had no such differences with 1.03 ± 0.06 mg/dl in the pre-, 0.91 ± 0.02 mg/dl in the 6th week, and 1.12 ± 0.022 mg/dl in the 12th week (Figure 3(b)). However, no significant differences were seen in urea and creatinine, either between the groups (as per the ANOVA test) or in the pre-, 6th week, and 12th week, between the groups in the Bonferroni test (*p* ≤ 0.01) (Tables 1 and 2).

### 3.4. Effects of Resistance Exercises on Leptin, Adiponectin, Interleukin-6, and Testosterone Levels

**Figure 4:**
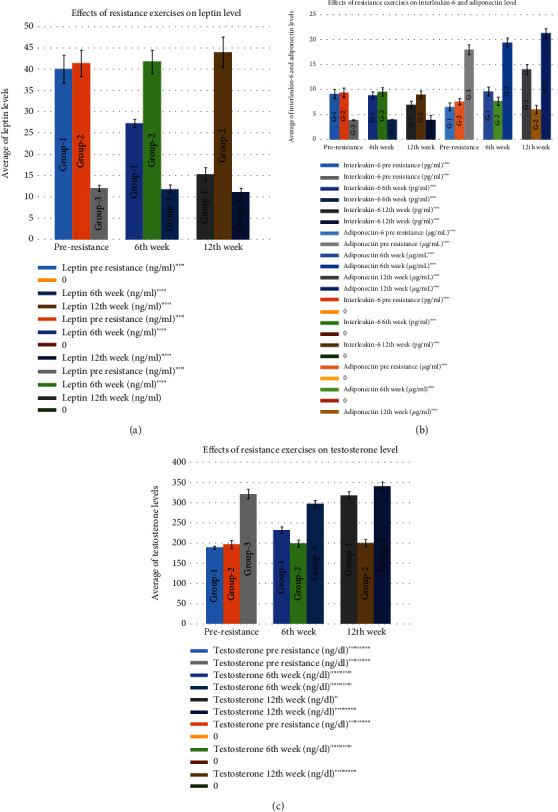
Effect of resistance exercises on hormone profile: (a) leptin; (b) interleukin-6 and adiponectin; (c) testosterone.

Leptin was seen to decrease from 40.02 ± 3.33 ng/ml in the preresistance group to 15.32 ± 1.56 ng/ml in the 12th week. However, no significant change was seen in groups 2 and 3 when comparing the preresistance values to the 12th week (for group 2) 41.47 ± 3.09 ng/ml in the pre-, 41.77 ± 2.79 ng/ml in the 6th week, and 44.05 ± 3.56 ng/ml in the 12th week; for group 3, 12.10 ± 0.70 ng/ml in the pre-, 11.85 ± 0.97 ng/ml in the 6th week, and 11.18 ± 0.89 ng/ml in the 12th week (Figure 4(a)). Adiponectin was seen to increase from 6.57 ± 0.82 *μ*g/ml (in the preresistance group) to 14.07 ± 0.97 *μ*g/ml (in the 12th week) in group 1. However, no such increase was noted in group 2 (7.63 ± 0.63 *μ*g/ml in the preresistance group to 6.08 ± 0.85 *μ*g/ml in the 12th week) and group 3 (18.04 ± 0.96 *μ*g/ml in the preresistance group, 19.43 ± 0.99 *μ*g/ml in the 6th week, and 21.3 ± 0.94 *μ*g/ml in the 12th week) (Figure 4(b)). Interleukin-6 decreased from 9.15 ± 0.90 pg/ml (in the preresistance group) to 7.00 ± 0.67 pg/ml in the 12th week (for group 1). However, no such developments were seen in group 2 (9.40 ± 0.91 pg/ml in the pre-, 9.56 ± 0.85 pg/ml in the 6th week, and 9.00 ± 0.75 pg/ml in the 12th week) and also in group 3 (3.93 ± 0.08 pg/ml in the pre-, 3.99 ± 0.07 pg/ml in the 6th week, and 3.93 ± 0.03 pg/ml in the 12th week) (Figure 4(b)). Testosterone was seen to increase from 189.44 ± 3.97 ng/dl (in the preresistance group) to 318.48 ± 9.61 ng/dl (in the 12th week) in group 1. But no such increase was seen in group 2 (197.44 ± 9.97 ng/dl in the preresistance group to 200.69 ± 8.90 ng/dl in the 12th week) and also in group 3 (321.85 ± 11.16 ng/dl in the preresistance group to 341.53 ± 10.11 ng/dl in the 12th week) (Figure 4(c)). Biochemical comparison between groups also showed many dissimilarities as depicted in Figure 4(c).

A significant difference is seen in adiponectin, leptin, and testosterone between the groups in the ANOVA test (Table 1). A significant change in leptin, adiponectin, interleukin, and testosterone values has been seen between preresistance and 12th week (Bonferroni test; *p* ≤ 0.01 (Table 2)).

## 4. Discussion

In this research, the consequences of a disciplined and well-organized resistance exercise program were scrutinized on body constitution, lipids, lipoproteins, liver and parameters, kidney, and other biochemical parameters. Consequently, it was decided that resistance isometrics modify the composition of the body. In many studies done in this area, resistance exercises are seen to have beneficial outcomes on body conformation in aged women with obesity of the sarcopenic type as envisaged by many researchers. It is accentuated that three months of integrated exercises (resistance+aerobic) appertained to middle-aged obese persons ushered positive upgrades in body conformations [20].

The glucose metabolic weakening in diabetes type 2 is a consequence of modification of various signaling pathways adjusting the uptake of glucose constituting insulin and exercise-enkindled signaling pathways. But during exercise, glucose utilization is near the normal range [21, 22], indicating possibly relevant signaling pathways modifying exercise-induced glucose utilization. Exercise-invigorating signal transduction can reinstate glucose metabolism in muscles resistant to insulin through both immediate triggerings of glucose transport and by bettering the sensitivity of insulin for up to 2 days after exercise. This is in accordance with our study, where we found glucose differences almost negligible during this short-term study.

In the course of a spell of exercise, the escalating contraction-motivating glucose utilization is connected to escalations in AMP-activated protein kinase (AMPK), which emanates in the phosphorylation of the Rab-GTPase-activating protein “tre-2/USP6, BUB2, cdc16 domain family member 1” (TBC1D1), which is associated with regulation of GLUT4 translocation in skeletal muscle.

It emerges that a somewhat dissimilar path is utilized to maintain glucose utilization at rest and entail TBC1 Domain Family, Member 4 (TBC1D4, also known as AS160), the TBC1D1 paralogue. TBC1D4 is entangled in the insulin-requiring regulation of glucose transporter type 4 (GLUT4) transfers and glucose utilization in myocytes and adipocytes. Insulin encourages the phosphorylation of TBC1D4 provoking its inactivation and as a result increases GLUT4 enterprise. TBC1D4 is also entangled in the maintenance of glucose utilization after exercise, by which elevation is interconnected with the increase in intracellular kinase Akt, the consequence of which is the phosphorylation of TBC1D4 [23]. However, in contradiction to TCB1D1, TCB1D4 seems to exhibit a retarded reciprocation to contraction/exercise inducement, with its inactivation endeavouring a result after exercise than during conduction of exercise. Exercise training may also have a good outcome such as chronic augmentations in TBC1D4 phosphorylation and thereby elevate basal serum insulin [24]. Repeated exercise bouts (exercise training) have been exhibited to elevate GLUT4 amount in populations with type 2 diabetes mellitus (T2DM) and metabolic syndrome (MetS) [25], and these elevations are correlated with changes in serum insulin [26]. Such rectifications are specific for a specific tissue, as exercise seems to boost skeletal muscle but not liver serum insulin, nor insulin-triggered glucose utilization in adipose tissue. Therefore, if the exercises would have continued for a longer time, we would have seen more changes in insulin levels.

The reciprocation of the lipid profile towards aerobic exercise has been scrutinized in detail in several researches. However, the response of resistance training has not been properly evaluated in height. It has been shown in many researches that endurance and resistance exercises exert influence on those whose basic levels of cholesterol, triglycerides, and LDL are higher, and the basic level of HDL is lower [27]. These results are similar to our study, which shows that total cholesterol, LDL, and triglycerides decreases with increased exercise and HDL increases with increased exercise. Goldberg et al. studied a single group of men and women. They reported depletion of the lipid profile. In most studies, remarkably, serum triglycerides were not influenced by resistance exercises in persons having high-intensity resistance training. Most of the research studies showed that HDL was increased with resistance training [28–30].

It has been seen that athletes mostly have higher urea levels, which is mainly an after-effect of stress training continuously. Urea amounts are also generally elevated after the accomplishment of poststress exercises (PSE) and may continue as such in the increased state for about 36 hrs postexercise. An elevation in the urea amount may be interconnected to a decline in the blood flow to the kidney (and glomerular filtration rate) subservient to fluid volume insufficiency, escalated protein breakdown, and/or rarely haemorrhage into the intestine, all of which may happen after PSE [30]. All these show that exercise elevates the short-time urea or creatinine but not the long-term elevation. Creatinine amount also generally gets elevated after PSE [31].

Contemporary research studies have depicted that fat in the abdominal region is integrated with a lowered state of health and inferior inflammation nondependent upon the BMI. These show that inactivity, without gaining weight, ushers in the build-up of visceral adipose tissue, while exercise training decreases visceral adipose tissue weight [32]. Recently, a central role for IL-6 in training-induced loss of visceral adipose tissue mass was reported. In a randomized placebo-controlled trial, abdominally obese adults were randomized to tocilizumab (IL-6 receptor antibody) or placebo during a 12-week intervention with either bicycle exercise or no exercise.

Most research has stated that plasma adiponectin facilitates the increase in insulin sensitivity by the mechanism of fatty acid oxidation in the liver, skeletal muscles, and blood vessels. Insulin sensitivity is also said to be activated by exercise training like ours, by the mechanism of enhancing adiponectin and increasing the level of 5-adenosine monophosphate kinase. Many research articles have noted that the level of adiponectin increases dramatically with the increase in insulin resistance. In our case, adiponectin increased from 6.57 *μ*gm/ml to 14.07 *μ*gm/ml after twelve weeks of resistance training in group 1. But in the case of group 2, there was no increase. In the case of group 3, there was a mild increase which could be considered insignificant. These coincide with the finding of other researchers mentioned [33, 34].

Hypoxia also exerts influence upon and makes changes in the adipocytes in their promulgation and liberation of adipokines, such as leptin, which plays a major part in the pronunciamento of the metabolism of energy. Group 3 showed an insignificant reduction in this case. In some studies, exposure to hypoxia led to a decline in body weight and also elevated the expression of leptin in adipose tissue. Hypoxia also led to an elevation in the expression of leptin in the adipose tissue of humans in vitro and cultured human adipocytes [35]. There are many conditions that could influence the final consequences, such as the degree and time span of hypoxia and participant characteristics. Several researches inclusive of physically fit participants have depicted that long-term exercise, such as cycling or athletics, led to depleted leptin levels immediately following exercise. Those results could be justified by the modifications in the disseminations and passage of foods in the adipocytes, which transpire in the course of perpetuated physical exercise. The reshufflings impede the emission of leptin from the adipocytes with an accompanying decline of its plasma levels [36, 37].

Testosterone, a steroid hormone, is created from cholesterol through a series of reactions. In our study, we found that there was a noteworthy increase in testosterone in group 1 only (increased from 189.44 ng/dl to 318.48 ng/dl). Most researchers have shown that circulating total testosterone and free testosterone are elevated after resistance exercise in men and comes back to the original level after some time. The conflicting results of most of the researches continue; others have not perceived any noteworthy difference [38, 39]. While it has been divulged that serum testosterone quantity declines in durability trained subjects, the few obtainable findings at hand regarding the outcomes of resistance tutelage on promulgating testosterone amounts are disputable.

## 5. Conclusions

We have seen that exercise helps in reducing lipid profile and decreases the cardiovascular and other risk factors when conducted in obese patients in high-altitude regions. Since very few studies have been done in this regard, it will help the physicians in prescribing the correct medications or exercises in this aspect. To date, very few studies have been done in high-altitude regions, taking into account so many parameters. Also, the unique resistance exercise provides a very good insight into the study. Parameters like adipokines and testosterone have been utilized to a good extent by this research at high altitude, and the correlations between them in this unique resistance exercise routine has been beautifully depicted. Also, obese people can take appropriate measures to enhance their health issues. The importance of adipokines and testosterone have been firmly established in this research which can help future generations.

This research has furnished new awareness into the physical health of people living in the high-altitude region of Abha, Kingdom of Saudi Arabia. If we consider the strengths of the research, one of them is the comparatively big sample size which was randomly chosen from various parts of Abha, implying that this particular sample may portray the entire population. However, the research has a number of limitations which must be accepted. The cross-sectional characteristic of the data does not permit the establishment of firm causal links. Moreover, the body fat percentage was not directly measured, which could lead to inconstant data in terms of the precise assessment of an adult's body composition and the affiliation of the groups.

## Figures and Tables

**Table 1:** Comparison of the significant levels of the biochemical markers among the groups.

ANOVA			
	*F*	Sig.	Decision
Glucose	1.024	0.360	NS
Insulin	14.086	0.000	S
Cholesterol	26.239	0.000	S
HDL	30.030	0.000	S
Triglyceride	16.119	0.000	S
LDL	19.153	0.000	S
Urea	0.764	0.466	NS
Creatinine	3.100	0.346	NS
Leptin	8.188	0.000	S
Adiponectin	28.706	0.000	S
Interleukin	126.393	0.000	S
Testosterone	14.771	0.000	S

*p* < 0.05 is considered significant (S) while *p* > 0.05 is considered not significant (NS).

**Table 2:** Comparison of the significant levels of the biochemical markers in the obese receiving resistance training exercise at different time intervals.

Post hoc analysis—multiple comparisons								
Bonferroni								
Dependent variable	(*I*) Time	(*J*) Time	Mean difference (*I*‐*J*)	Std. error	Sig.	95% confidence interval	
						Lower bound	Upper bound
Glucose	Preresistance	6th week	1.667	1.336	0.638	-1.54	4.88
		12th week	1.644	1.336	0.657	-1.56	4.85

Insulin	Preresistance	6th week	3.111	8.561	1.000	-17.45	23.67
		12th week	40.817^∗^	8.561	0.000	20.26	61.38

Cholesterol	Preresistance	6th week	49.989^∗^	8.360	0.000	29.91	70.07
		12th week	54.606^∗^	8.360	0.000	34.53	74.68

HDL	Preresistance	6th week	-4.200^∗^	1.323	0.005	-7.38	-1.02
		12th week	-10.200^∗^	1.323	0.000	-13.38	-7.02

Triglyceride	Preresistance	6th week	36.722	13.130	0.016	5.19	68.25
		12th week	74.544^∗^	13.130	0.000	43.01	106.08

LDL	Preresistance	6th week	26.583^∗^	7.955	0.003	7.48	45.69
		12th week	49.183^∗^	7.955	0.000	30.08	68.29

Urea	Preresistance	6th week	0.000	0.706	1.000	-1.69	1.69
		12th week	0.756	0.706	0.854	-0.94	2.45

Creatinine	Preresistance	6th week	-0.02556	0.02231	0.439	-0.1091	-0.0020
		12th week	-0.02811	0.02231	0.625	-0.0817	0.0255

Leptin	Preresistance	6th week	3.628	1.733	0.110	-0.53	7.79
		12th week	7.011^∗^	1.733	0.000	2.85	11.17

Interleukin	Preresistance	6th week	0.579	0.309	0.185	-0.16	1.32
		12th week	4.518^∗^	0.309	0.000	3.78	5.26

Adiponectin	Preresistance	6th week	-1.35111	0.59558	0.071	-2.7814	0.0792
		12th week	-4.40444^∗^	0.59558	0.000	-5.8347	-2.9742

Testosterone	Preresistance	6th week	-1.40167	0.61362	0.068	-2.8753	0.0719
		12th week	-3.32167^∗^	0.61362	0.000	-4.7953	-1.8481

^∗^Mean difference is significant at the *p* ≤ 0.01 level.

## Data Availability

The data used to support the findings of this study are available from the corresponding author upon request.
